# Ocular Migraine With Amaurosis Fugax of the Left Eye: A Case Report

**DOI:** 10.7759/cureus.28272

**Published:** 2022-08-22

**Authors:** Faria Tazin, Harendra Kumar, Muhammad A Israr, Camille Celeste Go

**Affiliations:** 1 Internal Medicine, East Liverpool City Hospital, East Liverpool, USA; 2 Surgery, Dow University of Health Sciences, Karachi, PAK; 3 Research, Larkin Community Hospital Palm Springs Campus, Hialeah, USA; 4 Family Medicine, Larkin Community Hospital Palm Springs Campus, Hialeah, USA

**Keywords:** internal carotid artery, atherosclerosis, loss of vision, amaurosis fugax, ocular migraine

## Abstract

In amaurosis fugax, there is a sudden loss of vision in one eye. Patients gain their vision after a few minutes. The most common underlying cause is atherosclerosis of the internal carotid artery. Other underlying causes include vasospasm of the internal carotid artery, which leads to hypoperfusion and is seen in vasculitis, ocular migraines, and systemic lupus erythematosus. In this case study, a 44-year-old male with a past medical history of migraine with aura presented to the emergency room with transient vision loss in the left eye, which lasted for two minutes. A computed tomography scan of the brain was negative for stroke. The patient was treated with intravenous fluid, aspirin, and enoxaparin sodium and instructed to follow up with neurology. A medical emergency such as amaurosis fugax caused by ocular migraine must be managed aggressively, and prompt imaging is necessary to exclude other causes.

## Introduction

Among adults, sudden, transient loss of vision in one eye, also known as amaurosis fugax (AF), is associated with internal carotid artery atherosclerosis and can portend a stroke or vasospasm of the artery [1]. AF can also be caused by ocular migraine [2]. As a recurrent neurological disorder, ocular migraines usually manifest as attacks of temporary reversible neurological symptoms lasting between five and 20 minutes. There is a mix of positive and negative symptoms of auras, including visual, sensory, and linguistic manifestations [3]. It has been reported that ischemic optic neuropathy and permanent arcuate scotomas may occur as a result of cerebral and ocular migraine [4]. This is a case report on a 44-year-old male with a past medical history of migraine with aura who suffered an abrupt unilateral vision loss while watching television that came back after three minutes. He presented to the ER and neurology was consulted. Magnetic resonance angiography (MRA) of the head and neck with/without contrast followed by magnetic resonance imaging (MRI) of the orbit and brain with/without contrast was performed, which showed no signs of acute intracranial abnormalities or vascular occlusions.

## Case presentation

A 44-year-old male with a past medical history of ocular migraines presented to the emergency room (ER) for painless, transient vision loss in his left eye, which lasted for two minutes. The patient stated that he was watching television when he felt a black curtain covering his left eye. The patient stated that he has had blurry vision in the past due to the aura associated with his ocular migraine but never had a complete loss of sight. The patient denied any other associated symptoms, any previous history of myocardial infarction, cerebral vascular accident, or any relevant family history.

On physical examination, the patient reported vision changes, was tachycardic at 101 beats per minute, had a blood pressure of 154/95 mmHg, respiratory rate of 16 breaths per minute, and oxygen saturation of 99% on room air. Laboratory examinations showed a decrease in the international normalized ratio to 1.0. The rest of the laboratory findings were unremarkable. No acute intracranial pathology was identified on the brain's CT scan. The patient was diagnosed with AF of the left eye with stroke-like symptoms due to a rare complication of ocular migraine. The patient was treated with intravenous fluid, aspirin, and enoxaparin sodium.

**Figure 1:**
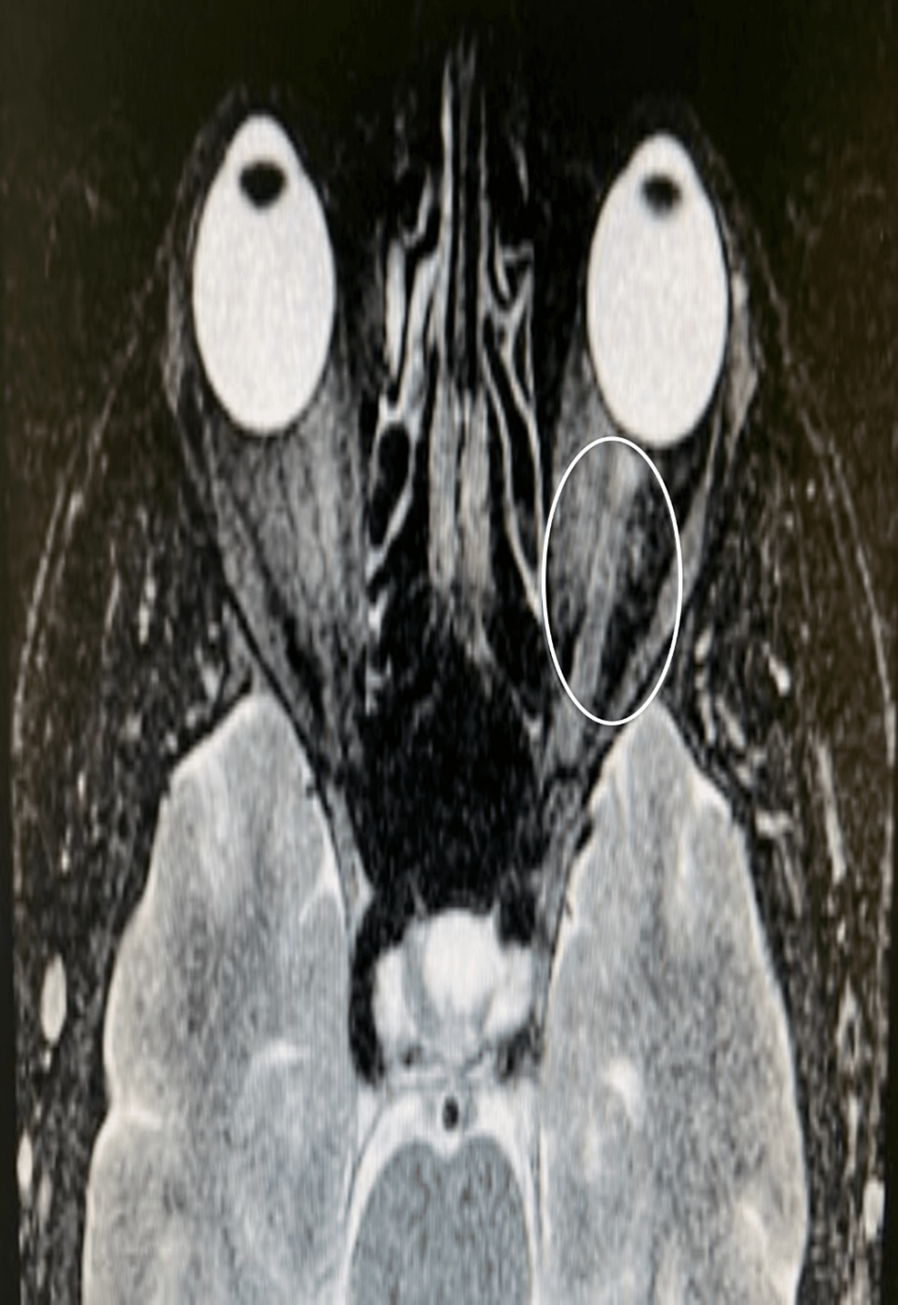
No signs of ischemia in the ophthalmic artery (circle)

**Figure 2:**
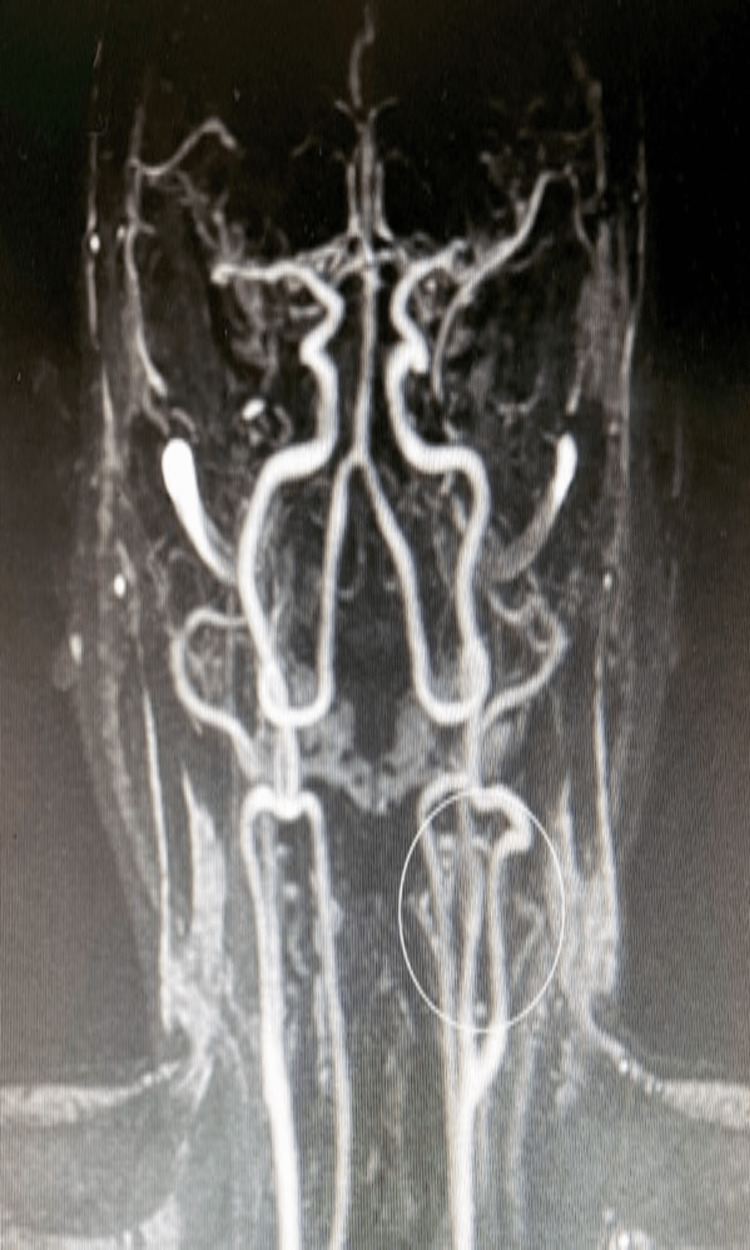
No signs of ischemia in the internal carotid artery (circle)

**Figure 3:**
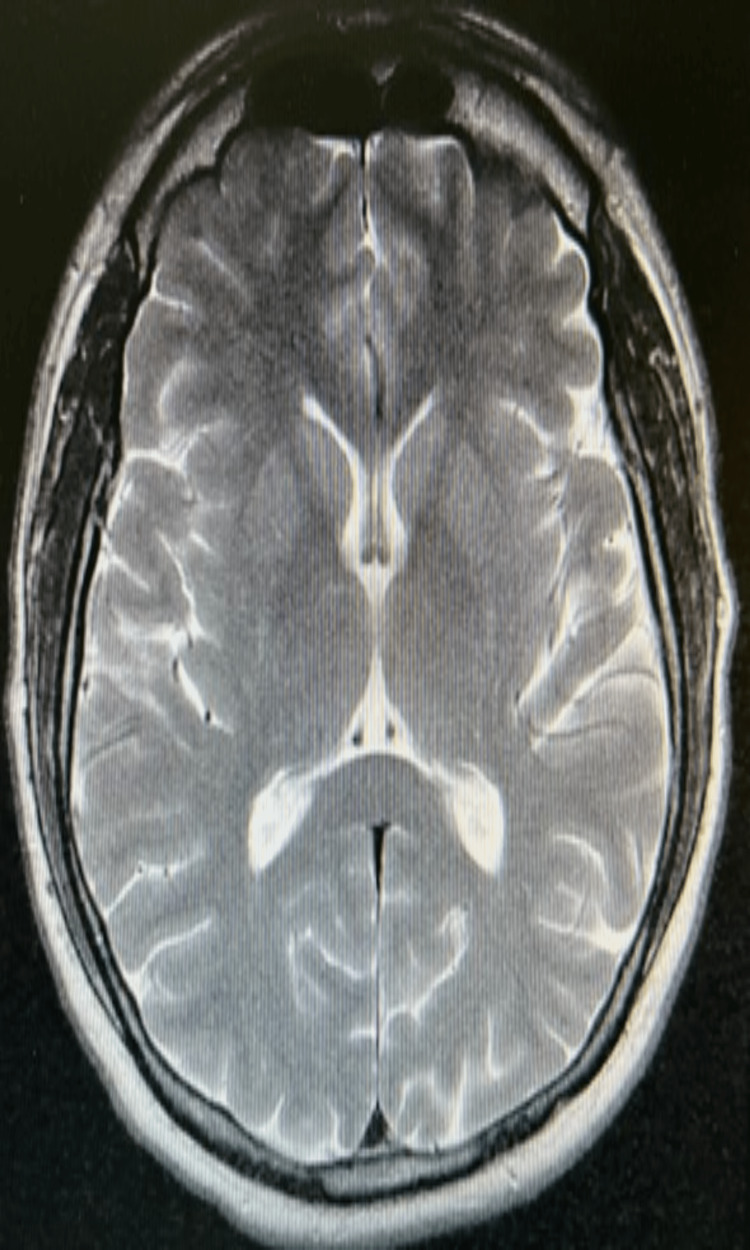
MRI of the brain was negative for thrombus and hemorrhage

Teleneurology was consulted and recommended MRA of the head without contrast, MRI of the orbit without contrast, MRI of the head/brain with and without contrast, and MRA of the neck with contrast. All imaging was unremarkable for any acute intracranial abnormalities or vascular occlusions. As shown in Figures 1-3 below, the CT, MRI, and MRA scans were unremarkable. They showed no signs of hemorrhage, thromboembolism, or atherosclerosis. On the third day of admission, the patient was discharged home in stable condition, with outpatient appointments with neurology and ophthalmology.

## Discussion

A particular kind of transient ischemic attack (TIA) known as AF exclusively affects the eye and results in brief blindness in one eye. A restriction in the ocular artery's normal flow is what causes the condition, which typically lasts a few minutes. However, it may also be brought on by thromboembolism from the carotid artery because of non-atherosclerotic reasons, such as aortic dissection or embolization, heart difficulties, or thrombosis of the retinal or optic nerve vessels. The same diagnostic technique as for any TIA is essential in AF-related conditions. Young people (aged 50 years or less) with ischemic strokes or TIA should be screened for carotid artery dissection (CAD), a prevalent cause of stroke in this age group [5]. CT and MRI scans should be done to rule out serious causes.

The most prevalent symptom is pain in the head, face, or neck, which affects 64-74% of patients, appears first in 60-70% of cases, and is the sole symptom in 2-5% of them. Of the patients, 9-26% have neck pain, 34-53% of patients complain of facial pain, and 66-78% of patients experience headaches. Due to the sympathetic system, which innervates the pupils and eyelids via a plexus closely linked to the internal carotid artery, partial Horner syndrome is seen in 28-41% of patients. Due to internal CAD's inability to influence sudomotor fibers, which follow the external carotid artery, it is only partial. Of the cranial nerves IX to XII, the hypoglossal nerve is the one that is damaged most commonly. It runs from the hypoglossal canal to the angle of the jaw, exactly opposite the lateral side of the internal carotid artery's extracranial portion. Cranial nerve palsy has been reported in 8-16% of cases. Ocular, facial, and trigeminal motor nerves may also be impacted. CADs must be identified and treated as soon as possible since about 75% of CADs are the cause of ischemia episodes. Cerebral infarction (80-84%), TIA (15-16%), AF (3%), ischemic optic neuropathy (4%), and retinal infarction (all ischemic events) (1%) are all ischemic events [6].

We want to emphasize that having coronary artery disease does not preclude the use of intravenous thrombolysis, which is equally effective in treating acute ischemic stroke [7]. Following coronary artery disease, antiplatelet therapy and anticoagulation are both used to prevent further strokes. Currently, there is no evidence to support the idea that one treatment is better than another [8]. Acute coronary artery disease therapy has included both surgical and endovascular methods. Surgical techniques include extracranial to intracranial bypass, carotid artery ligation or clipping, and thromboendarterectomy with patch angioplasty. The use of self-expanding stents and percutaneous balloon angioplasty during endovascular operations has virtually supplanted surgical methods [9]. The indications, effectiveness, and need for endovascular intervention are still uncertain since there has not been any randomized study comparing the available treatments. A migraine with an aura, on the other hand, is a persistent illness characterized by bouts of reversible localized neurological symptoms that normally last between five and 20 minutes. Typically, a headache with migraine-like symptoms appears after the aura symptoms. Though it is less common, auras may sometimes exist without giving a person a headache. Visual, sensory, and verbal symptoms of auras are common and may include both positive and negative symptoms. The physicians must be aware that another etiology may be at work since, in our instance, the headache began before the migraine, which is rare for migraine.

## Conclusions

AF presents with transient unilateral reversible vision loss. The most common cause is atherosclerosis of the internal carotid artery; however, other common causes include ocular migraine and vasculitis. A CT or MRI scan must be done promptly to rule out stroke and the patient must be referred to neurology. Patients with ocular migraines have vasospasm of the internal carotid artery, which leads to sudden reversible vision loss. The vasospasm causes hypoperfusion of the optic nerve, which can cause ischemic optic neuropathy and permanent arcuate scotomas. In this case report, the patient had a past medical history of migraine with an aura, which led to AF. MRI scans of the head/brain with and without contrast were negative, which shows that there was no permanent damage to the optic nerve. The patient was discharged on day three and he was instructed to follow up with neurology and ophthalmology to monitor his condition. If not treated promptly with medication, it can cause permanent vision loss in one eye. However, antiplatelet, anticoagulation, and calcium channel blockers may be needed depending on the root cause, to prevent ocular migraine-related severe complications like AF.
